# Psychische Gesundheit von LGBT*-Jugendlichen in Österreich: Scoping Review und Forschungsagenda auf Basis internationaler Evidenz

**DOI:** 10.1007/s40211-022-00436-x

**Published:** 2022-11-01

**Authors:** Magdalena Siegel, Christiana Nöstlinger, Flo Dürrauer, Stefanie Kirchner, Thomas Niederkrotenthaler, Martina Zemp

**Affiliations:** 1 Institut für Klinische und Gesundheitspsychologie, Universität Wien, Wien, Österreich; 2 Department of Public Health, Institute of Tropical Medicine, Antwerpen, Belgien; 3 Abteilung für Sozial- und Präventivmedizin, Medizinische Universität Wien, Wien, Österreich

**Keywords:** Sexuelle und geschlechtliche Minderheiten, Systematische Übersichtsarbeit, Österreich, Psychische Gesundheit, Jugendliche, Sexual and gender minorities, Systematic review, Austria, Mental health, Adolescent

## Abstract

Eine Vielzahl internationaler Studien zeigt, dass lesbische, schwule, bisexuelle, transgender und andere Jugendliche mit diversen sexuellen Orientierungen und/oder Geschlechtsidentitäten (LGBT*-Jugendliche) psychische Vulnerabilitäten, aber auch spezifische Ressourcen aufweisen. Es ist jedoch unklar, inwieweit diese Ergebnisse auf Jugendliche in Österreich übertragbar sind, da sich die soziolegalen und entwicklungsbezogenen Kontexte zwischen Ländern unterscheiden. Wir haben gemäß PRISMA-Richtlinien ein systematisches Scoping Review durchgeführt, um (1) publizierte Studien zur psychischen Gesundheit von LGBT*-Jugendlichen in Österreich zu identifizieren und darauf aufbauend (2) Forschungsempfehlungen abzuleiten, die durch internationale Evidenz ergänzt werden. Es wurden fünf wissenschaftliche Datenbanken (PsycInfo, PSYNDEX, PubMed, Scopus, Web of Science; März 2022) systematisch durchsucht und zusätzlich Expert_innen aus Forschung und Community kontaktiert, um einschlägige Studien zu finden. Es konnten nur zwei veröffentlichte empirische Studien zur psychischen Gesundheit von LGBT*-Jugendlichen in Österreich gefunden werden, was die geringe Studienlage in Österreich reflektiert. Vor diesem Hintergrund skizzieren wir eine detaillierte Forschungsagenda nach einem sozio-ökologischen Ansatz. Die Einbeziehung der sexuellen Orientierung und nicht-binärer Geschlechtsidentitäten in populationsbasierten Studien zur Untersuchung von Erstauftreten, Prävalenz und Verlauf psychischer Belastungen sowie die gezielte, ressourcenorientierte und entwicklungssensitive Forschung auf allen Ebenen scheinen vorrangig, um gesundheitliche Ungleichheiten und gesellschaftliche Stigmatisierung zu verringern und LGBT*-Jugendliche in ihrer Entwicklung bestmöglich zu unterstützen.

## Abstract (en)

A robust body of international evidence documents that lesbian, gay, bisexual, transgender, and other youth with diverse sexual orientations and/or gender identities (i.e., sexual and gender minority youth) face unique mental health vulnerabilities but are also equipped with unique resources. However, it is unclear to what extent these findings are applicable to sexual and gender minority youth in Austria, because the sociolegal and developmental contexts differ across countries. According to PRISMA guidelines, we conducted a systematic scoping review (1) to identify published studies on the mental health of sexual and gender minority youth in Austria, and, based on this, (2) to derive research recommendations supplemented by international evidence. We searched five scientific databases (PsycInfo, PSYNDEX, PubMed, Scopus, Web of Science; March 2022) and additionally contacted researchers and community leaders to find pertinent studies. Only two published empirical studies on the mental health of sexual and gender minority youth in Austria could be located, reflecting the sparse state of research in Austria. Against this background, we outline a detailed research agenda following a socio-ecological approach. Including sexual orientation and non-binary gender identities in population-based studies to assess onset, prevalence, and trajectory of mental health burdens, as well as conducting targeted, resource-based, and developmentally sensitive research on all levels seem paramount to reduce health disparities and societal stigma and to support sexual and gender minority youth in their development.

Die europäische Agentur der Grundrechte (FRA) führte 2019 die bislang größte Erhebung (*N* = 139.799; nicht repräsentativ) zu Diskriminierungserfahrungen von LGBTI-Personen^1^ in Europa durch [1]. Deskriptive Ergebnisse dieser Studie zeigen, dass LGBTI-Jugendliche in Österreich (15–17 Jahre; *n* = 119) durch ihre stigmatisierte Identität erheblichen minderheitenspezifischen Belastungssituationen ausgesetzt sind (Abb. 1), die vom Geheimhalten der eigenen Identität sowie Diskriminierung im familiären und schulischen Umfeld bis hin zu Gewalterfahrungen reichen [1]. Basierend auf internationalen Befunden [2, 3] und theoretischen Annahmen [4, 5] ist davon auszugehen, dass diese Stressoren zu einer erhöhten psychischen Belastung führen. LGBT*-Jugendliche in Österreich sind also potenziell vulnerabel in Bezug auf ihre psychische Gesundheit. Eine systematische Synthese einschlägiger empirischer Befunde liegt bis dato jedoch nicht vor.

**Figure 1:**
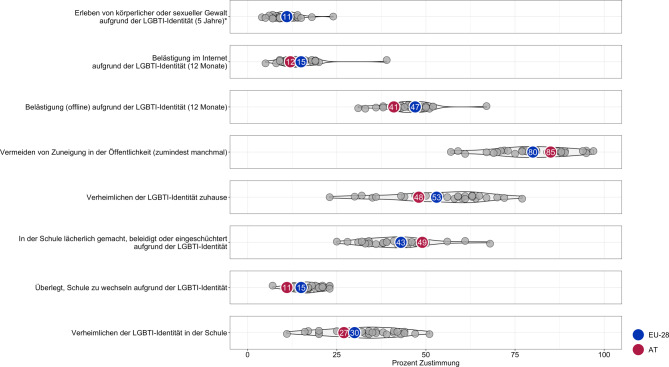

## Deutschsprachige und internationale Evidenz

Die wenigen bisherigen Studien zur psychischen Gesundheit von LGBT*-Jugendlichen aus Deutschland [6, 7] und der Schweiz [8] deuten übereinstimmend mit den Ergebnissen der FRA auf eine erhöhte psychische Belastung gegenüber heterosexuellen, cisgender^2^ Gleichaltrigen sowie dem Erleben minderheitenspezifischer Stressoren hin. Eine rezente Studie mit LGBT*-Jugendlichen aus dem deutschsprachigen Raum fand eine höhere psychische Belastung für transgender Jugendliche im Vergleich mit cisgender LGB*-Jugendlichen; die Rate an Suizidversuchen im vergangenen Jahr (zwischen 10 % für cisgender männliche Jugendliche und 28 % für transgender Jugendliche) ist als alarmierend hoch einzustufen [9]. Ältere österreichische Studien mit LGB-Erwachsenen identifizierten retrospektiv berichtete Viktimisierung in der Jugend als Prädiktor für Suizidalität [10].

**Figure 2:**
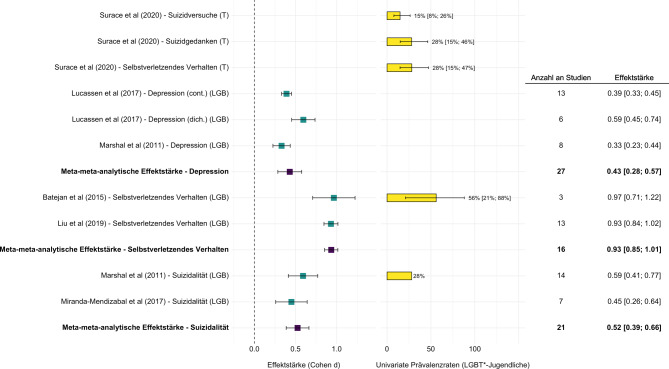

Auch international weisen LGBT*-Jugendliche, verglichen mit heterosexuellen, cisgender Gleichaltrigen, eine erhöhte Vulnerabilität für verschiedene psychische Belastungen (z. B. Depressivität, Ängstlichkeit oder Suizidalität) auf (siehe Abb. 2 sowie [11]). Prospektive Studien zeigen ein ontogenetisch frühes Auftreten (ab 10 Jahren) dieser erhöhten Belastung und über die weitere Adoleszenz bis ins junge Erwachsenenalter bestehende, wenn auch variierende Verläufe [12].

## Das Minderheitenstressmodell im Entwicklungskontext

Das prominenteste Modell zur Erklärung der erhöhten psychischen Vulnerabilität von LGBT*-Populationen [14, 15] ist das Minderheitenstressmodell [4, 5]. Das Modell postuliert neben allgemeinen psychischen Risiko- und Schutzfaktoren zusätzliche, minderheitenspezifische Faktoren auf zwei Ebenen [4]: *Distal* wirken (i) strukturelle Faktoren (z. B. die rechtliche Situation für LGBT*-Personen [16]) und (ii) interpersonelle Diskriminierungs- oder Gewalterfahrungen [4]. Für die Betroffenen können diese distalen Stressoren *proximal* eine Internalisierung negativer gesellschaftlicher Einstellungen [4], eine erhöhte Empfindlichkeit gegenüber Zurückweisung [17] oder die Verheimlichung der LGBT*-Identität [4] auslösen. Viele minderheitenspezifischen Stressoren (z. B. soziolegales Klima, elterliche Zurückweisung) treten bereits in der Adoleszenz bzw. ab der späten Kindheit auf [18, 19]. Der Zusammenhang zwischen dem Erleben dieser Stressoren und psychischer Belastung ist bei LGBT*-Jugendlichen vielfach repliziert [2, 3].

Die pathogenetische Wirkung von Minderheitenstress erfolgt durch eine erhöhte Vulnerabilität gegenüber allgemeinen psychischen Risikofaktoren [20, 21]: Er führt u. a. zu maladaptiven kognitiven Prozessen (z. B. Rumination und Hypervigilanz aufgrund der Verheimlichung der LGBT*-Identität), emotionaler Dysregulation und fehlendem sozialen Rückhalt (z. B. durch ein stigmatisierendes familiäres Umfeld), die wiederum transdiagnostisch die psychische Gesundheit beeinträchtigen [20]. Entsprechend diesen Annahmen weisen LGBT*-Jugendliche konsistent höhere Raten an allgemeinen Risikofaktoren auf als heterosexuelle, cisgender Gleichaltrige [22].

Entwicklungsbedingte Spezifika und der soziolegale Wandel in den letzten Jahren bedingen eine separate Betrachtung gegenwärtiger Jugendlichenpopulationen in Abgrenzung zu sowohl LGBT*-Erwachsenen als auch früheren adoleszenten Kohorten [19, 23, 24]. Coming Out-Prozesse finden bei heutigen LGB-Jugendlichen in westlichen Ländern – u. a. bedingt durch höhere soziolegale Akzeptanz und Sichtbarkeit – früher statt als in älteren Kohorten [24]. Minderheitsspezifische Entwicklungsaufgaben fallen so in eine Phase markanter physiologischer und sozialkognitiver Veränderungen in der Adoleszenz. Diese ist generell eine vulnerable Phase für die Ausbildung von psychischen Störungen für Jugendliche [24]. Außerdem sind heutige LGBT*-Jugendliche geprägt von zentralen Sozialisations- und Entwicklungskontexten – allen voran Familie, Schule und sozialen Medien –, die eine wesentliche Quelle von minderheitenspezifischen Stressoren und Ressourcen darstellen [19, 23] und ihrerseits gesellschaftlichen Wandel durchlaufen.

## Das vorliegende Scoping Review

LGBT*-Jugendliche in Österreich sind eine potenziell vulnerable Population in Bezug auf ihre psychische Gesundheit. Bislang fehlt allerdings eine systematische Erhebung des diesbezüglichen Forschungsstands – und damit ein Gradmesser der Wahrnehmung dieser Population und ihrer Bedürfnisse. Eine national differenzierte Synthese ist zentral, da selbst Befunde aus Deutschland oder der Schweiz aufgrund zeitlich diskordanter soziolegaler Entwicklungen und Angebotsstrukturen für LGBT*-Populationen nur eingeschränkt übertragbar sind [25].

Dieses Scoping Review hat entsprechend zwei Ziele: Erstens erfolgt eine systematische Synthese der Forschung zur psychischen Gesundheit von LGBT*-Jugendlichen in Österreich. Zweitens sollen darauf basierende Forschungsempfehlungen abgeleitet werden, ergänzt durch internationale Evidenz.

## Methoden

Die Methodik des Scoping Reviews wurde aufgrund der Neuheit des Forschungsgebiets und der erwarteten Studienheterogenität gewählt [26]. Die Konzeption und Durchführung erfolgte gemäß PRISMA-ScR-Richtlinien [26] und kann im Detail dem präregistrierten Studienprotokoll entnommen werden, ebenso wie die Onlinematerialien 1–5 (https://osf.io/vdgy2/).

### Einschlusskriterien und Suchstrategie

Tab. 1 zeigt die Einschlusskriterien des vorliegenden Reviews. Die systematische Literatursuche (04.–06.06.2020; Updates: 09.04.2021; 08.03.2022) erfolgte in fünf wissenschaftlichen Datenbanken (PsycInfo, PSYNDEX, PubMed, Scopus, Web of Science). Die Suchstrategie kombinierte Sets mit deutschen und englischen Suchbegriffen zu (1) LGBT*, (2) Kindern und Jugendlichen und (3) Österreich und wurde durch datenbankspezifische Filter optimiert (siehe Onlinematerial 2). Auf Suchbegriffe zur psychischen Gesundheit wurde aufgrund der intendierten Breite des Reviews verzichtet. Stattdessen wurden Abstracts bzw. Volltexte individuell auf diesbezügliche Einschließbarkeit geprüft. Zusätzlich wurden Interessensgruppen im deutschsprachigen Raum sowie Expert_innen in Österreich kontaktiert, um nach weiteren unbekannten Studien bzw. Daten zu fragen, die die Einschlusskriterien erfüllen (Liste auf Anfrage).

**Table 1:** 

Typ	Einschlusskriterium
Studientyp	1. In Fachzeitschrift (mit Peer-Review) publizierte qualitative, quantitative oder mixed methods Studie
	2. Publikationsjahr > 1999
Population	1. Alter: Mittleres oder Medianstichprobenalter < 18 Jahre bzw. Maximalalter von 25 Jahren (Einschluss bei konfligierenden Kriterien; Ausschluss von retrospektiven Studien mit Erwachsenen)
	2. LGBT*-Status: Nicht-heterosexuelle Selbstdefinition: erhoben mittels Selbstberichtitems zu Identität, Verhalten oder sexuelle Anziehung; Transgender-Identität: erhoben mittels Selbstberichtitems oder klinischer Diagnostik von Transsexualität (ICD) bzw. Geschlechtsdysphorie (DSM)
Kontext	1. Studiendurchführung in Österreich (> 50 % der Teilnehmenden zum Erhebungszeitpunkt in Österreich lebend) bzw. Bericht getrennter Ergebnisse für österreichische Substichproben (Ausschluss bei unklarer Stichprobenkomposition)
Konzept	1. Erfassung mindestens eines Maßes für die psychische Gesundheit/Belastung oder für allgemeine und minderheitenspezifische psychische Risiko- und Schutzfaktoren

### Studienauswahl

Titel und Abstracts der Suchresultate wurden einer Vorauswahl (Erstautorin) anhand der definierten Einschlusskriterien (Tab. 1) unterzogen. Die dabei ausgeschlossenen Studien wurden durch ein_e Ko-Autor_in [FD] unabhängig auf Einschließbarkeit überprüft. Die anschließende Volltextprüfung erfolgte unabhängig durch dieselben zwei Autor_innen (Interrater-Reliabilität: 100 %; κ = 1,0).

### Datenextraktion

Folgende Informationen wurden aus der Primärliteratur extrahiert (Doppelkodierung Erstautorin) und in tabellarischer Form zusammengefasst: Methodik; Datenerhebungsort und -zeitraum; Stichprobentyp, -größe (*N*), -zusammensetzung bzgl. Alter, sexueller Orientierung, Geschlechtsidentität, Ethnie/Migrationshintergrund; Forschungsfragen/-ziele; Ergebnisse (lt. Autor_innen); Limitationen (lt. Autor_innen). Wie bei der Durchführung von Scoping Reviews üblich [26], führten wir keine systematische Bewertung der methodischen Qualität der Primärstudien durch, weil die sondierende Zusammenfassung existierender Studien mit besonderer Berücksichtigung von Forschungslücken im Zentrum stand.

## Ergebnisse

**Figure 3:**
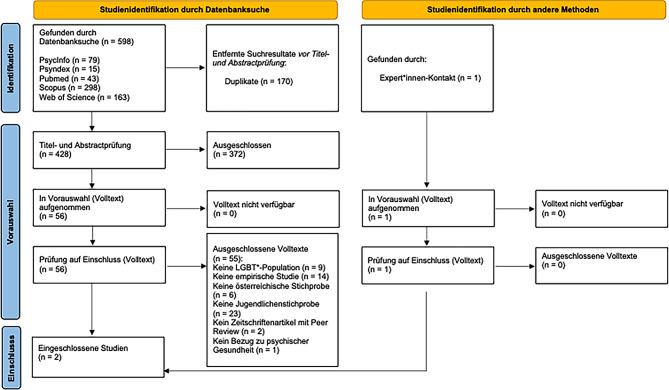

Abb. 3 zeigt die Studienauswahl (Referenzen ausgeschlossener Studien bei Volltextprüfung siehe Onlinematerial 3). Zwei Studien [9, 27] erfüllten die Einschlusskriterien (Tab. 2; Onlinematerial 4). Beide Studien waren quantitativ mit rezenten Datenerhebungsperioden (2015–2020 [27] bzw. 2020 [9]) [27]. verglichen eine Inanspruchnahmepopulation der Spezialambulanz für Geschlechtsdysphorie in Innsbruck bzw. Hall in Tirol (*N* = 69) bezüglich verschiedener Maße der Identitätsentwicklung mit einer deutschsprachigen Normstichprobe. Die Ergebnisse zeigten eine insgesamt der Norm entsprechenden, allerdings in Substichproben bzw. Subskalen auffällige Identitätsentwicklung. [9] untersuchten mittels randomisiert kontrolliertem Experiment (on site und online) die Wirkung von LGBT*-spezifischen Suizidpräventionsvideos auf LGBT*-Jugendliche und junge Erwachsene (*N* = 483) in Österreich (55 % der Stichprobe) und Deutschland. Es zeigte sich eine kurzfristige, kleine Verbesserung hinsichtlich Suizidgedanken bei transidenten Jugendlichen sowie hinsichtlich Hilfesuchabsichten in der Gesamtstichprobe. Weitere Merkmale, Ergebnisse und Limitationen der zwei inkludierten Studien sind in Tab. 2 aufgeführt.

**Table 2:** 

	Studie	
Studienmerkmal	Haid-Stecher et al. 2020 [27]	Kirchner et al. 2022 [9]
Methodik	Quantitativ	Quantitativ
*N*	69	483
Datenerhebungsort und -zeitraum	Österreich (Tirol); 2015–2020	Österreich (Wien und online; 54,66 %); Deutschland (online; 43,89 %); andere Länder (nicht spezifiziert; online; 1,45 %); 2020
Stichprobenbeschreibung	Inanspruchnahmepopulation der Spezialambulanz für Geschlechtsdysphorie an der Universitätsklinik für Kinder- und Jugendpsychiatrie, Psychotherapie und Psychosomatik Innsbruck bzw. Hall in Tirol (Einschluss: mind. Erstgespräch und Fragebogendiagnostik). Diagnostik: Transsexualismus (F64.0) bzw. Störung der Geschlechtsidentität des Kindesalters (F64.2) nach ICD-10 durch Kinder- und Jugendpsychiater_in	Anfallsstichprobe (on site in Wien; ansonsten online) von LGBT*-Jugendlichen und jungen Erwachsenen; Rekrutierung über LGBT*-Organisationen, Influencer_innen, Social Media
Stichprobenzusammensetzung^a^	Alter: *M* = 15,6; *SD* = 1,3; ab 12 Jahren	Alter: *M* = 18,96, *SD* = 2,24 (Interventionsgruppe); *M* = 19,16, *SD* = 2,25 (Kontrollgruppe); Range: 14–22 Jahre
	Sexuelle Orientierung: n. b.	Sexuelle Orientierung: 27,54 % bisexuell, 24,22 % schwul, 21,74 % lesbisch, 14,49 % queer, 4,97 % questioning, 4,35 % pan-/omnisexuell, 2,69 % asexuell
	Geschlechtsidentität: Zuweisungsgeschlecht bei Geburt: 76,8 % weiblich; 23,2 % männlich; Selbstidentifikation: 76,8 % Transjunge; 20,3 % Transmädchen; 2,9 % nicht-binär	Geschlechtsidentität: 52,38 % cisgender weiblich, 26,92 % cisgender männlich, 20,7 % transgender/nonbinary
	Ethnie/Migrationshintergrund: n. b.	Ethnie/Migrationshintergrund: n. b.
Forschungsfragen/-ziele	Vergleich der Identitätsentwicklung (i. S. v. Beeinträchtigungen des Funktionsniveaus) bei transidenten Jugendlichen mit deutschsprachiger Normstichprobe	Wirkung von *It Gets Better* Suizidpräventionsvideos (mittels RCT) auf Suizidgedanken (primäres Outcome); hilfesuchendes Verhalten in Bezug auf Suizidgedanken, Identitätsherausforderungen/negative Identität, Stimmung, Hoffnungslosigkeit (sekundäre Outcomes); Prüfung moderierender Effekte von Geschlechtsidentität, sexueller Orientierung, depressiven Symptomen; Prüfung mediierender Effekte von Identifikation mit dem/der Video-Protagonist_in
Hauptbefunde (Autor_innen)	Gesamtstichprobe im Mittel unauffällig (definiert als T < 60) im Vergleich zur Normierungsstichprobe (T = 56,5; Gesamtskala); *ns* Unterschied zwischen Transjungen und -mädchen	Gesamtstichprobe: Kein signifikanter Interventionseffekt von *It Gets Better* Suizidpräventionsvideos zu T2 (nach Exposition; *d* = −0,06) oder T3 (4 Wochen Follow-Up; *d* = −0,04)
	Identitätsdiffusion in klinisch auffälligem Bereich (T > 60; Gesamtskala) bei 36 % der Stichprobe	T2: Kleiner und positiver Interventionseffekt bezüglich hilfesuchendem Verhalten (*d* = 0,09); *ns* zu T3 (*d* = 0,03)
		T2: Kurzfristiger Rückgang (nach Exposition) der Suizidgedanken bei nichtbinären/transgender Personen in der Interventionsgruppe im Vergleich zur Kontrollgruppe im Ausmaß eines kleinen Effekts (*d* = −0,10), *ns* zu T3 (*d* = −0,05); stärkere Wirkung bei nichtbinären/transgender Personen mit schwerer depressiver Symptomatik
		Mediation: Indirekter und kleiner positiver Effekt durch Identifikation mit Protagonist_in (T2)
Limitationen (Autor_innen)	Mittlere Fallzahl	Eingeschränkte Generalisierbarkeit der Stimuli
	Keine Kontrolle für Ausmaß der körperlichen und sozialen Transition	Erhebung kurzzeitiger Effekte bei einmaliger Exposition
	Selbsturteil ohne zusätzliche fremdanamnestische Beurteilung	Keine stratifizierten Analysen bezüglich Zuweisungsgeschlecht bei Geburt oder Ethnie möglich
		Substanzieller LTFU von Online-Teilnehmenden (T3)
		Niedrige interne Konsistenz für Skala zur Erhebung des hilfesuchenden Verhaltens

*n. b.* nicht berichtet, *RCT* Randomized controlled trial, *ns* nicht signifikant, *LTFU* Loss to follow up (Verlust der Nachbeobachtung)

^a^Einschluss von Kirchner et al. (2022) bei konfligierenden Alterskriterien (*M* > 18 Jahre; oberes Alterslimit < 25 Jahre; siehe Tab. 1 und Studienprotokoll)

## Diskussion

Die Forschung zur Epidemiologie von psychischen Störungen im Kindes- und Jugendalter in Österreich sowie zur hiesigen Versorgungssituation in diesem Bereich ist allgemein spärlich (mit nennenswerten Ausnahmen, z. B. [28, 29]). Besonders markant erscheint dieser Forschungsmangel jedoch bei LGBT*-Populationen: Das nahezu (d. h. mit zwei Ausnahmen) völlige Fehlen von österreichischen Studien zur psychischen Gesundheit von LGBT*-Jugendlichen unterstreicht gerade angesichts der potenziellen Vulnerabilität dieser Population den dringenden Forschungsbedarf. Die rezenten Datenerhebungsperioden sowie weitere, nicht unseren Einschlusskriterien entsprechende Arbeiten der beiden Forschungsgruppen (z. B. [30, 31]) zeigen allerdings, dass die Lebensrealitäten von LGBT*-Jugendlichen in der österreichischen Forschungslandschaft zunehmend Aufmerksamkeit erhalten.

Beide inkludierten Studien berücksichtigten LGBT*-spezifische Herausforderungen und Ressourcen in österreichischen Versorgungsstrukturen: [27] durch die Betrachtung einer Inanspruchnahmepopulation einer österreichischen Spezialambulanz für transidente Kinder und Jugendliche, [9] durch die Evaluation niederschwelliger, community-basierter und spezifischer Interventionsangebote für LGBT*-Jugendliche [9]. Als sekundäres Ergebnis weisen wir zudem auf die hohe psychische Belastung von transidenten Jugendlichen hin [9], die es zu replizieren gilt. Insgesamt fehlt inklusive, populationsrepräsentative und bestenfalls längsschnittliche Grundlagen- und Interventionsforschung zur psychischen Gesundheit von LGBT*-Jugendlichen in Österreich sowie eine systemische Betrachtung ihrer Entwicklungskontexte.

### Forschungsempfehlungen

**Figure 4:**
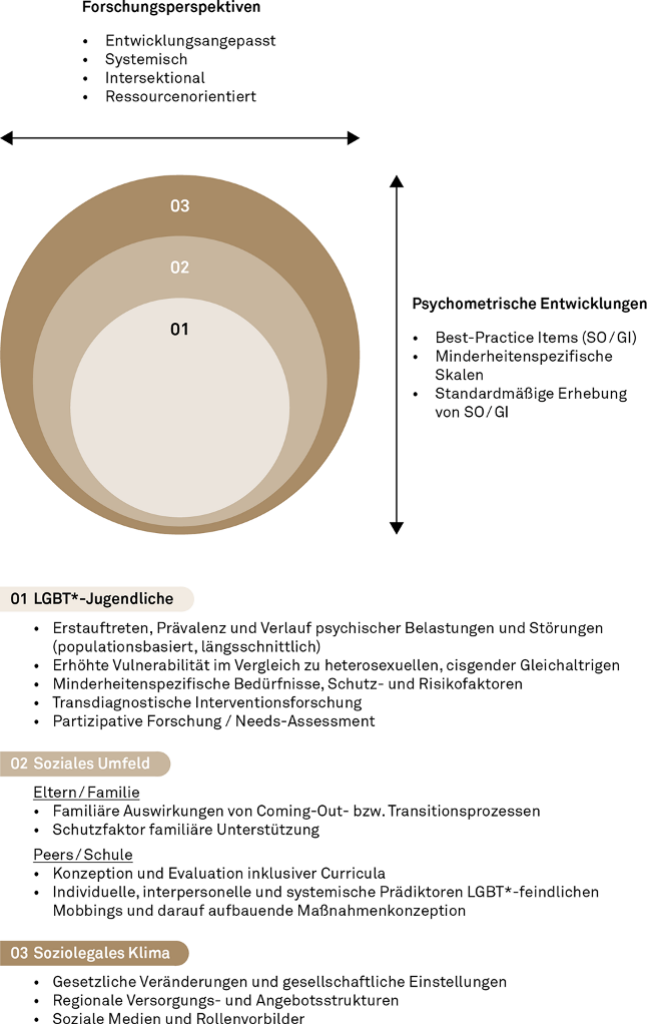

Abb. 4 gibt Forschungsempfehlungen zu LGBT*-Jugendlichen in Österreich basierend auf internationaler Evidenz sowie einem sozio-ökologischen Ansatz [19, 32]. In Onlinematerial 5 finden sich weiterführende Erläuterungen und Erklärungen dieser Forschungsempfehlungen und -perspektiven (u. a. zur Methodik künftiger Studien der Grundlagen- und Interventionsforschung, zur routinemäßigen Erfassung diverser Geschlechtsidentitäten in künftigen populationsbasierten Erhebungen, Berücksichtigung relevanter Subgruppen).

#### LGBT*-Jugendliche

Vorrangig und in Einklang mit nationalen [33] und internationalen Forderungen [34] sind die Erhebung populationsbasierter, längsschnittlicher Daten bezüglich Erstauftreten, Prävalenz und Verlauf psychischer Belastungen bei LGBT*-Jugendlichen sowie die Ermittlung einer potenziellen, durch Minderheitenstress erklärbaren, Vulnerabilität verglichen mit heterosexuellen, cisgender Gleichaltrigen. Wir empfehlen die Erhebung von sexueller/romantischer Orientierung und transgeschlechtlichen Erfahrungen in allgemeinen, populationsbasierten Studien mit Jugendlichen (z. B. [35]). Zudem benötigt es Subgruppenvergleiche innerhalb der LGBT*-Populationen, um besonders vulnerable Gruppen (z. B. transidente Jugendliche [9]) zu identifizieren. Individuelle Forschungsgruppen können die Forderung nach Repräsentativerhebungen nur schwer umsetzen, da finanzielle und personelle Ressourcen fehlen. Insbesondere minderheitenspezifische Entwicklungsaufgaben sowie Schutz- und Risikofaktoren [19] können allerdings auch in Anfallsstichproben kostengünstig (z. B. durch Online-Erhebungen) untersucht werden (siehe Onlinematerial 5).

Wichtig ist zudem die Entwicklung und Evaluation psychologisch-psychotherapeutischer Interventionen für LGBT*-Jugendliche (auch international rar bzw. unzureichend evaluiert [36]). Transdiagnostische Präventions- und Interventionsprogramme, die die Reduktion von Minderheitenstress als zentrale Determinante einer erhöhten psychischen Vulnerabilität Betroffener zum Ziel haben, erscheinen erfolgsversprechend [21]. Die Einbindung Jugendlicher und Community-Vertreter_innen in die Studienkonzeption durch partizipative Ansätze [37] ist zentral.

#### Soziales Umfeld

Familie, Schule und Peers sind für (LGBT*-)Jugendliche zentrale Entwicklungskontexte, die wesentlich zur Reduktion bzw. Verstärkung minderheitenspezifischer Vulnerabilitäten beitragen und kompensatorische Wirkung entfalten können [19, 23]. Die Familie ist eine zentrale Ressource [38], die es in Behandlung, Beratung und familientherapeutischer Interventionsforschung einzubeziehen gilt. Diesbezügliche Forschung mit LGBT*-Jugendlichen fehlt nahezu völlig [38]. Zudem ist die Konzeption und Evaluation von inklusiven Lehrplänen sowie die Erhebung von Prädiktoren LGBT*-feindlichen Mobbings [19] indiziert.

#### Soziolegales Klima

Zahlreiche gesetzliche Änderungen der letzten Jahre (z. B. Einführung der Ehe für gleichgeschlechtliche Paare, neue Geschlechtseinträge) haben in Österreich zu einer höheren rechtlichen Sicherheit von LGBT*-Personen geführt. Gesellschaftliche Stereotypien bestehen allerdings weiterhin [39] und Versorgungs- und Angebotsstrukturen variieren regional beträchtlich. US-amerikanische Studien zeigen den Einfluss eines sich wandelnden soziolegalen Klimas [40] sowie regionaler Angebote [41] auf die psychische Gesundheit LGB(T)*-Jugendlicher. Dies sollte – insbesondere durch das österreichische Stadt-Land-Gefälle bei Angeboten für LGBT*-Personen – gemeinsam mit den Auswirkungen sozialer Medien und Rollenvorbilder [31] auch in der österreichischen Forschung Berücksichtigung finden.

#### Forschungsperspektiven

Forschung zu und mit LGBT*-Jugendlichen soll *entwicklungsangepasst* allgemeine und minderheitenspezifische Entwicklungsaufgaben berücksichtigen. Daran knüpft eine *systemische* Sichtweise, die Jugendliche als in verschiedenen zentralen Systemen (Familie, Schule, Peers) eingebunden [19] sieht. Zusätzliche Diversitätskategorien (z. B. Ethnie/Migrationshintergrund) bzw. deren verschränkte Wirkung sind unter einer *intersektionalen* Perspektive zu beachten. Letztens bedarf es eines *ressourcenorientierten* Blicks auf die Förderung von minderheitenspezifischen Schutzfaktoren.

#### Psychometrische Entwicklungen

Die Umsetzung o. g. Forschungsempfehlungen basiert u. a. auf einer psychometrisch fundierten, altersgerechten und sprachlich sensiblen Entwicklung von (i) Items zu sexueller Orientierung und Geschlechtsidentität, (ii) Skalen zu minderheitenspezifischen Konstrukten und (iii) Indizes zur Quantifizierung regionaler Unterschiede in Angebotsstrukturen für LGBT*-Jugendliche. Unter Einbezug von Jugendlichen entwickelte und für populationsbasierte sowie Anfallsstichproben einsetzbare, valide Skalen sind für den deutschsprachigen Raum derzeit nicht verfügbar. Sexuelle Orientierung und (nicht-binäre) Geschlechtsidentität sollten standardmäßig erhoben werden, auch um für mögliche Konfundierungen (z. B. zur psychischen Belastung) zu kontrollieren.

### Limitationen

Erstens wurden nur Studien eingeschlossen, die in Fachzeitschriften mit Peer-Review publiziert wurden. Zukünftige, auf graue Literatur ausgerichtete Synthesen sollten durch mehrstufige Suchstrategien (z. B. Universitätsdatenbanken, Community-Kontakt, EU-Institutionen) diese Limitation aufgreifen und mögliche Gründe für die Übertragungslücke in Fachpublikationen thematisieren. Zweitens konnten wir durch unsere Suchstrategie keine Studien identifizieren, in denen LGBT*-bezogene Variablen und/oder Ergebnisse zu einer österreichischen Substichprobe zwar analysiert, aber nicht explizit ausgewiesen wurden.

### Konklusion

LGBT*-Jugendliche in Österreich sind potenziell psychisch belastet und müssen sich minderheitenspezifischen Stressoren und Entwicklungsaufgaben stellen. Das vorliegende Scoping Review offenbart die vorhandenen Forschungslücken. Es bedarf dringend zielgerichteter, ressourcenorientierter, populationsbasierter, nach Subgruppen stratifizierter und entwicklungssensitiver Grundlagen- und Interventionsforschung, um gesundheitliche Unterschiede und gesellschaftliche Stigmatisierung zu reduzieren und LGBT*-Jugendliche in ihrer Entwicklung zu unterstützen.

## Footnotes

1 LGBTI = Lesbian, gay, bisexual, trans, intersex. Der vorliegende Beitrag fokussiert auf lesbische, schwule, bisexuelle und transgender (LGBT*)-Jugendliche. Der Asterisk (*) bezeichnet eine beliebige Erweiterung auf andere Begriffe, die Personen zur Selbstbeschreibung ihrer sexuellen/romantischen Orientierung oder Geschlechtsidentität verwenden.

2 Personen, bei denen Geschlechtsidentität und Zuweisungsgeschlecht bei Geburt übereinstimmen.
